# Muscle motor point identification is essential for optimizing neuromuscular electrical stimulation use

**DOI:** 10.1186/1743-0003-11-17

**Published:** 2014-02-25

**Authors:** Massimiliano Gobbo, Nicola A Maffiuletti, Claudio Orizio, Marco A Minetto

**Affiliations:** 1 Department of Clinical and Experimental Sciences, University of Brescia; Laboratory of Neuromuscular Rehabilitation, Clinic “Domus Salutis”, Brescia, Italy; 2 Neuromuscular Research Laboratory, Schulthess Clinic, Zurich, Switzerland; 3 Division of Endocrinology, Diabetology and Metabolism, Department of Medical Sciences, University of Turin, Turin, Italy

**Keywords:** Muscle motor point, Motor entry point, Discomfort, Motor unit recruitment, Evoked muscle tension

## Abstract

Transcutaneous neuromuscular electrical stimulation applied in clinical settings is currently characterized by a wide heterogeneity of stimulation protocols and modalities. Practitioners usually refer to anatomic charts (often provided with the user manuals of commercially available stimulators) for electrode positioning, which may lead to inconsistent outcomes, poor tolerance by the patients, and adverse reactions. Recent evidence has highlighted the crucial importance of stimulating over the muscle motor points to improve the effectiveness of neuromuscular electrical stimulation. Nevertheless, the correct electrophysiological definition of muscle motor point and its practical significance are not always fully comprehended by therapists and researchers in the field. The commentary describes a straightforward and quick electrophysiological procedure for muscle motor point identification. It consists in muscle surface mapping by using a stimulation pen-electrode and it is aimed at identifying the skin area above the muscle where the motor threshold is the lowest for a given electrical input, that is the skin area most responsive to electrical stimulation. After the motor point mapping procedure, a proper placement of the stimulation electrode(s) allows neuromuscular electrical stimulation to maximize the evoked tension, while minimizing the dose of the injected current and the level of discomfort. If routinely applied, we expect this procedure to improve both stimulation effectiveness and patient adherence to the treatment.

The aims of this clinical commentary are to present an optimized procedure for the application of neuromuscular electrical stimulation and to highlight the clinical implications related to its use.

## Introduction

**Figure 1:**
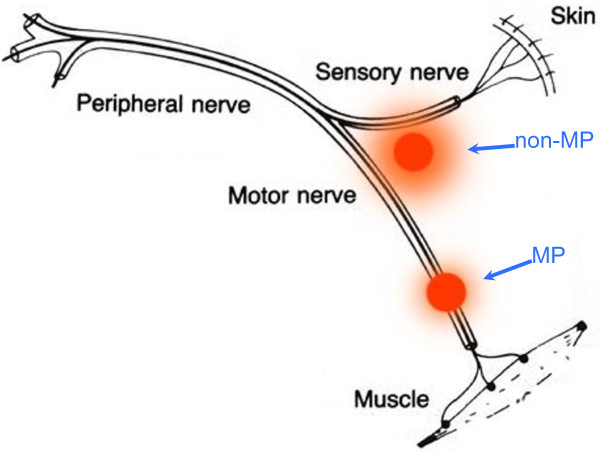
**Schematic representation of a mixed peripheral nerve and two stimulation sites.** When the active electrode precisely overlies the motor point (MP), less current is required to excite the motor axons and thus to elicit the muscle contraction. Alternatively, stimulation on the other site (non-MP) requires higher current intensity to reach the motor branch, with possible excitation of the sensory fibers conveying pain.

Transcutaneous neuromuscular electrical stimulation (NMES) involves the application of electrical stimuli to superficial skeletal muscles, with the main objective to trigger visible and valid muscle contractions due to the activation of motor neuron axons or intramuscular axonal branches [1]. It is largely adopted in rehabilitation practice to restore or preserve muscle mass and function in case of prolonged periods of disuse/immobilization [2], and it is also receiving increasing attention as a preoperative strengthening intervention (i.e. “prehabilitation”) [3]. Nevertheless, the technique presents some inherent limitations that foster a lack of general consensus on NMES effectiveness and utility in clinical practice. The three main limitations of NMES are: 1) considerable discomfort; 2) limited spatial recruitment that results in low evoked tension and early occurrence of fatigue; 3) poor control of dosage. These limitations are partly due to non-optimal application of NMES by the end-users, who frequently place electrodes in poorly effective locations as recently outlined by Doucet et al. [4]. Specifically, the practitioner should consider that benefits are strictly modality- and dose-dependent and, as a consequence, rigorous methods are crucial for optimal NMES delivery. In this view, muscle motor point (MP) identification prior to placement of stimulation electrodes represents a simple, inexpensive and straightforward strategy to improve NMES use in the context of clinical rehabilitation. In fact, the position of the stimulation electrodes critically influences the pathway of the spreading current and therefore its relative density through the different anatomical structures within the current field, namely sensory and motor branches of the peripheral nerve. As depicted in Figure 1, stimulation via motor points is likely to involve chiefly motor branch excitation, while non-optimal electrode positioning would require higher current levels to reach and excite the motor branch with concomitant greater excitation of pain afferent fibers. For this reason, the proper placement of stimulation electrodes over the identified MP(s) allows to overcome, at least in part, two of the previously described NMES limitations, namely discomfort and limited spatial recruitment.

With reference to updated evidence-based knowledge and through a reappraisal of key physiological concepts, aims of this commentary are to present a patient-tailored approach for optimizing NMES delivery in superficial skeletal muscles, namely through individual MP identification, and to highlight the clinical implications related to its use.

### Basic concepts

Skeletal muscle MP is the location of the skin area above the muscle in which an electrical pulse applied transcutaneously evokes a muscle twitch with the least injected current. In other words, it represents the skin area above the muscle where the motor threshold is the lowest for a given electrical input [5-7].

**Figure 2:**
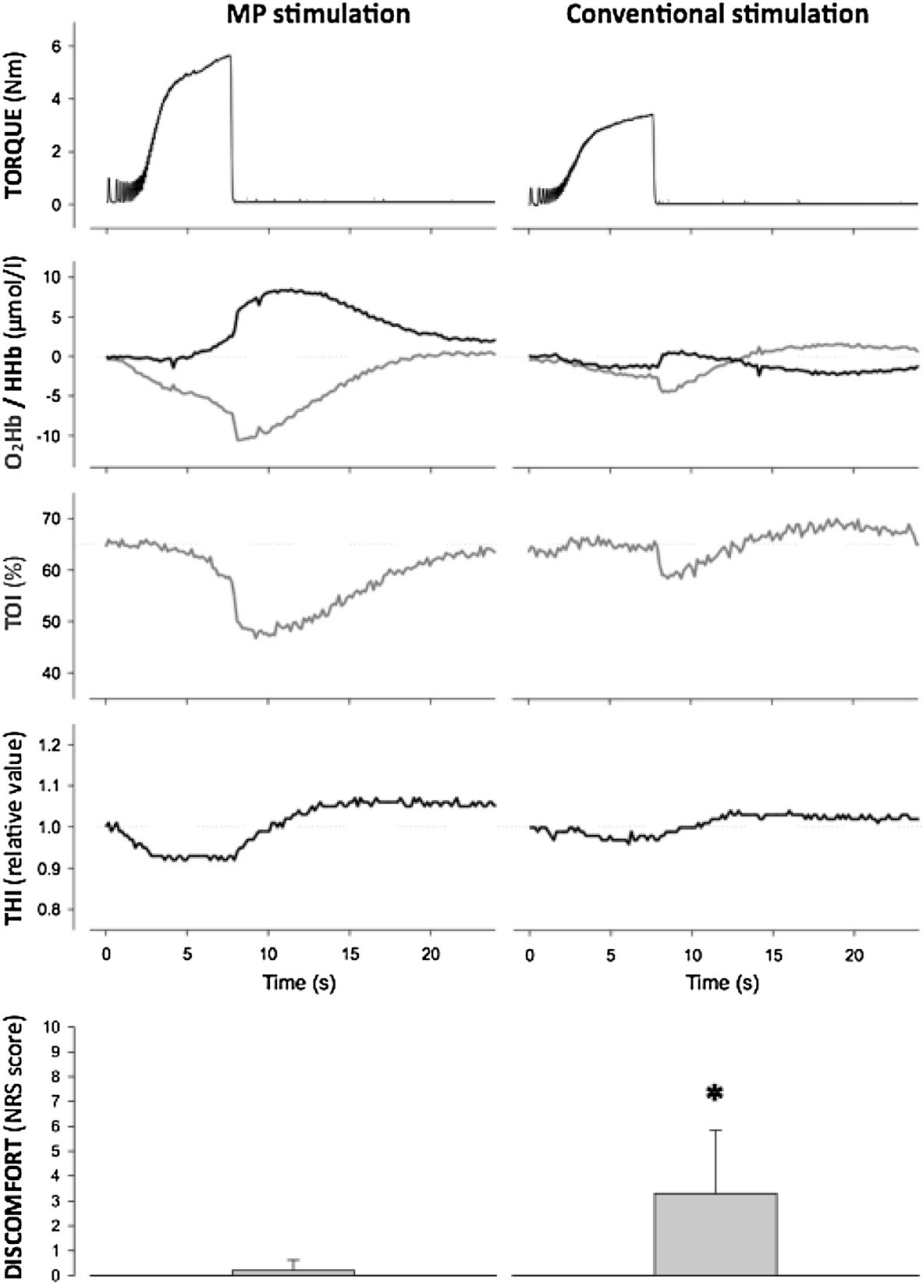
**Torque traces and oxygenation/deoxygenation profiles during neuromuscular electrical stimulation and recovery.** The signals, recorded from a representative subject during a frequency ramp contraction (from 2 to 50 Hz in 7.5 s) and the subsequent recovery phase, refer to tibialis anterior muscle stimulation via the motor point (MP stimulation) and stimulation following anatomic reference charts for electrode placement (conventional stimulation). Note that MP stimulation results in greater mechanical stress and metabolic demand than conventional stimulation. O_2_Hb = oxyhemoglobin; HHb = deoxyhemoglobin; TOI = tissue oxygenation index; THI = total hemoglobin index. The bottom panels are related to subject perception of discomfort evaluated with a numeric rating scale (NRS) and show group mean ± SD values for the two conditions studied in 10 healthy subjects: MP stimulation induces significantly (* P < 0.05) less discomfort than conventional stimulation. NRS scores: 0 = no discomfort; 10 = maximum discomfort. (Modified from Gobbo et al. [6]. Copyright © 2011 Springer. Used with permission provided by Copyright Clearance Center, license number: 2913660233993).

**Figure 3:**
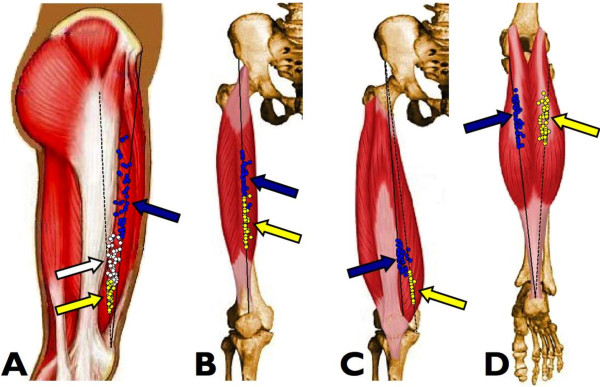
**Position of the muscle motor points for the quadriceps and gastrocnemii in 53 healthy subjects.** The arrows indicate the average motor point (MP) positions along the respective reference lines. **A)** Vastus lateralis muscle MPs (blue circles, proximal MP; white circles, central MP; yellow circles, distal MP). The continuous black line is the reference line for the proximal MP, while the dashed black line is the reference line for the central and distal MPs. **B)** Rectus femoris muscle MPs (blue circles, proximal MP; yellow circles, distal MP). **C)** Vastus medialis muscle MPs (blue circles, proximal MP; yellow circles, distal MP). The continuous black line is the reference line for the proximal MP, while the dashed black line is the reference line for the distal MP. **D)** Medial (blue circles) and lateral (yellow circles) gastrocnemii muscle MPs. (Modified from Botter et al. [5]. Copyright © 2011 Springer. Used with permission provided by Copyright Clearance Center, license number: 2923641294715).

Gobbo et al. [6] clearly evidenced that NMES delivered through individually identified MPs, as opposed to anatomical charts for electrode positioning, is critical to maximize the evoked muscular tension and the related metabolic changes while minimizing current intensity and discomfort (Figure 2). Moreover, Botter et al. [5] showed a large inter-individual variability of the muscle MP location in lower limb muscles (Figure 3). Together, these findings suggest that muscle MPs should be carefully searched prior to NMES delivery, thus pursuing a patient-tailored approach that accounts for the specific anatomical morphology of each individual. This approach is supposed to be an essential step in optimizing NMES delivery. Indeed, as demonstrated by Lieber and Kelly [8], the most important determinants of the tension evoked by NMES are not electrode size or stimulation current, or any other external controllable factor, but some intrinsic properties of the muscle, e.g., individual patterns of motor nerve branching.

The muscle MP definition relies on its electrophysiological identification and has to be distinguished from the anatomical definition of the motor entry point, which is actually the location where the motor branch of a nerve enters the muscle belly. The motor entry point is often confused with, and used as synonym for, the electrophysiologically-identified MP: this common misconception may induce end-users to adopt anatomical charts with topographical indications of “motor points” (which are actually motor entry points) as a guide for stimulation electrode positioning. As outlined above, available published charts presenting “anatomical motor points” are of limited value for administering NMES [6].

### Theory in practice

Based on the aforementioned notions, muscle MP locations cannot simply be inferred from marketed anatomical charts, but a specific electrophysiological procedure based on muscle surface mapping is required for precise MP identification.

**Figure 4:**
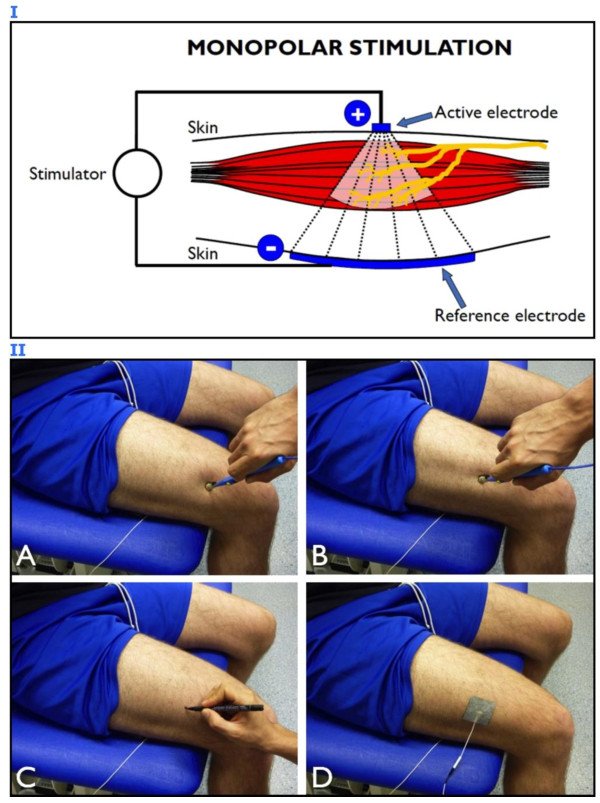
**Motor point identification procedure. Panel I:** schematic representation of monopolar stimulation. The active electrode is placed over the muscle region of interest and the reference electrode is placed over the antagonist muscle or opposite to the active electrode to close the stimulation current loop. **Panel II:** electrophysiological procedure for motor point (MP) identification and proper electrode placement. The skin surface above the vastus lateralis muscle is mapped with a pen-electrode, the dispersive reference electrode being placed opposite to the active one; the joint angle should be the one adopted for the subsequent stimulation protocol in order to avoid skin displacement with respect to the underlying neural and muscular structures. **A)** The muscle contractile response is not evident when the pen is not facing the MP area. **B)** The MP of the target muscle is identified as the specific site where a minimal mechanical response is generated with the lowest current intensity. **C)** The identified MP is marked with a felt tip. **D)** The active electrode is placed exactly over the identified MP.

The suggested procedure is feasible and quick: muscle MPs can be easily identified by scanning the skin surface with a commercially available stimulation pen-electrode (“active” or “negative” electrode with a surface of approximately 1 cm^2^) and with a second electrode (usually called “reference” or “dispersive” or “positive” or “return” electrode) – larger than the active electrode (around tens of square centimeters) – that is placed over the antagonist muscle or opposite to the active electrode (monopolar configuration) (Figure 4, panel I).

While stimulating at very low frequency (1 or 2 Hz) and intensity (starting from 1 mA), using a monophasic or biphasic wave lasting 100–200 μs for subjects without neurological problems (longer duration is usually required in case of denervation or paresis), the operator should slightly press the pen-electrode on a specific area of the skin overlying the target muscle for 3–5 seconds, then the pen-electrode is moved to adjacent locations to check for the presence of mechanical responses (“twitching”). If no location reacts to the chosen level of current, the stimulation amplitude is slowly increased (with steps of 1 mA) and skin scanning over the muscle surface is repeated until a clear contraction of the muscle is observed or, alternatively, when a mechanical response of its tendon is perceived by manual palpation. Thereafter, the stimulation current is decreased to a value providing a minimal twitching response that should be detectable only when the pen-electrode exactly faces the muscle MP. Eventually, the position of the identified MP is marked on the skin and adopted as the centroid of the stimulation electrode placement as represented in Figure 4, panel II (that illustrates the procedure of vastus lateralis MP mapping performed by an experienced operator – MAM – on the anterior thigh of a healthy subject – MG – who provided written informed consent for both execution of the procedure and image publication). The idea behind this procedure is to determine the skin area most responsive to electrical stimulation, that is the one where the stimulation electrode provides the greatest muscle response per current dose.

A remarkable consideration pertains to muscle length during skin mapping: Crochetiere et al. [9] showed that MPs do not remain in the same location but rather move by about 2–3 cm as the joint is flexed and extended (due to muscle lengthening/shortening). For this reason, muscle length should be the same during MP mapping and during the subsequent NMES session.

### Can the spatially fixed and incomplete recruitment be optimized?

NMES is usually delivered using one or more active electrodes positioned in the proximity to the MPs and one reference electrode closing the stimulation loop [2]. This electrode configuration implies that the recruitment of motor units is not only limited, but also spatially fixed [10,11]. Therefore, the same muscle units are repeatedly activated by the same amount of electrical current, which, in turn, hastens the onset of muscle fatigue [10,12]. Such early occurrence of fatigue represents a major limitation of NMES. In order to maximize the spatial recruitment during NMES, thus minimizing the extent of muscle fatigue, it has been recommended to adopt different tricks during a stimulation session such as altering the length of the stimulated muscle and/or displacing the active electrodes [2]. However, a change in the population of activated fibers could also be obtained through a multichannel stimulation technique. Malesević et al. [13] recently showed that asynchronous NMES delivered to the quadriceps of paraplegic patients via multiple electrodes (one dispersive electrode positioned at the distal part of the quadriceps and four active electrodes distributed over the quadriceps) delayed the occurrence of fatigue with respect to single-channel synchronous stimulation (one electrode positioned over the proximal and another over the distal part of the muscle). Besides its use for functional electrical stimulation applications, the asynchronous stimulation delivered through a “multi-path” system has recently been applied to quadriceps rehabilitation in patients after anterior cruciate ligament reconstruction [14,15] and in patients with moderate to severe knee osteoarthritis [16]. The multi-path NMES modality was superior to traditional NMES performed with two active electrodes positioned over the vasti MPs and one dispersive electrode closing the stimulation loop [14] and comparable to volitional resistance training [16] for the improvement of functional capacity and quadriceps strength. Although preliminary, these findings suggest that spatial recruitment can be maximized through a non-synchronous activation of different muscle volumes. The proper placement of stimulation electrodes over the MPs of different muscles (e.g., vastus lateralis and rectus femoris) or different muscle portions (vastus medialis obliquus and vastus medialis longus) within a muscle group is an obvious prerequisite for optimizing the effectiveness of the multi-path paradigm.

### Clinical perspectives

The advances attained in NMES application [15] are generating a comprehensive understanding of the mechanisms involved in its effectiveness that are still poorly translated to clinical practice [4]. Certainly, some of this evidence is not easy to access or difficult to integrate in the existing clinical framework. In clinical settings, a major problem with NMES is the incorrect placement of stimulation electrodes [4]. As outlined by Gobbo et al. [6], although the approximate position of MPs for different muscles can be easily found in a number of manuals, charts and atlases, electrode placement over the approximate MP position is likely to result in ineffective NMES use, mainly as a consequence of low evoked tension and concomitant discomfort. MP identification prior to NMES delivery, which is completed in less than a minute, is expected to have relevant clinical implications in terms of greater acceptability by patients (discomfort is a major limitation of NMES that may lead to dropouts or, at the other extreme, to excessive lowering of the stimulus amplitude with ineffective levels of muscle contraction), greater treatment effectiveness (earlier and better recovery), less adverse reactions (e.g., skin irritation), and eventually lower cost-effectiveness ratio compared to conventional NMES with no MP identification.

## Conclusions

Quick and accurate MP identification prior to NMES has the potential to minimize the amount of current injected to the muscle and thus to minimize the sensation of discomfort, while maximizing spatial recruitment and evoked muscle tension.

In light of the recent evidences, we expect the use of the standard patient-tailored approach described in this commentary to improve clinical practice by optimizing NMES application. Therefore, we recommend the systematic implementation of the MP mapping procedure into clinical applications of NMES to further attain beneficial outcomes.

## Abbreviations

NMES: Neuromuscular electrical stimulation; MP: Motor point.

## Competing interests

The authors declare that they have no competing interests.

## Authors’ contributions

MG and MAM conceived the commentary and drafted the manuscript. NAM and CO contributed to drafting the manuscript and provided critical revision. All authors read and approved the final manuscript.
